# Diet and risk for hernia: a Mendelian randomization analysis

**DOI:** 10.3389/fnut.2024.1265920

**Published:** 2024-06-18

**Authors:** Yanjiang Yang, Biao Han, Wenwen Yang

**Affiliations:** ^1^The People's Hospital of Qiandongnan Autonomous Prefecture, Kaili, Guizhou, China; ^2^Department of Thoracic Surgery, The First Hospital of Lanzhou University, Lanzhou, Gansu, China; ^3^Gansu Province International Cooperation Base for Research and Application of Key Technology of Thoracic Surgery, The First Hospital of Lanzhou University, Lanzhou, Gansu, China; ^4^The First Clinical Medical College, Lanzhou University, Lanzhou, Gansu, China

**Keywords:** Mendelian randomization, incisional hernia, dietary intake, umbilical hernia, inguinal hernia

## Abstract

**Background:**

The relationship between dietary factors and hernias is currently unclear.

**Methods:**

The UK Biobank was used to extract dietary factors that were used as exposures, including intake of alcohol, non-oily fish, beef, fresh fruit, oily fish, salad/raw vegetables, dried fruit, coffee, cereal, salt, tea, water, cooked vegetables, cheese, Lamb/mutton, pork, poultry, processed meat, and bread. The FinnGen biobank was used to obtain GWAS data on hernias as outcomes. The main analysis of this study was performed using the weighted median, MR-Egger, and IVW methods. Cochran’s Q test was utilized to assess heterogeneity. To find potential outliers, the MR-PRESSO method was used. Leave-one-out analysis was employed to assess the IVW method’s robustness.

**Results:**

Alcoholic consumption per week (OR: 0.614; *p* = 0.00614) reduced the risk of inguinal hernia. Alcohol intake frequency (OR: 1.309; *p* = 0.0477) increased the risk of ventral hernia (mainly including incisional hernia and parastomal hernia). The intake of non-oily fish (OR: 2.945; *p* = 0.0214) increased the risk of inguinal hernia. Salt added to food (OR: 1.841; *p* = 0.00267) increased the risk of umbilical hernia. Cheese intake (OR: 0.434; *p* = 0.000536) and dried fruit intake (OR: 0.322; *p* = 0.00716) decreased the risk of ventral hernia, while cooked vegetable intake (OR: 4.475; *p* = 0.0380) increased the risk of ventral hernia. No causal relationships were found with hernias from other dietary factors.

**Conclusion:**

Inguinal, umbilical, and ventral hernias are all related to dietary factors.

## 1 Introduction

Inguinal hernia, umbilical hernia, and ventral hernia (including incisional hernia and parastomal hernia) are the three types of hernias, according to the 10th revision of the International Classification of Diseases. Inguinal hernias are the most common hernias, and umbilical hernias are also frequent hernias (1). Incisional hernias occur after laparotomies at a rate of 5–20% and more than 30% in high-risk individuals (2). There have been many studies investigating risk factors for hernias (3–9), but few studies have analyzed the effects of dietary factors on hernias (10). Dietary factors are important factors affecting health and disease (11–14). Therefore, this study used the methods of Mendelian randomization (MR) to analyze the effect of dietary factors on hernias.

## 2 Methods

MR identifies the causal relationship between exposures and outcomes by employing genetic variations as instrumental variables (IVs). Three fundamental assumptions must be met for MR to function (15). First, there was no connection between the IVs and any probable confounding factors. Second, there must be robust correlations between the IVs and exposure variables. Third, there are no direct connections between the IVs and outcomes. As a result of using deidentified and freely accessible data from the IEU Open GWAS project, this study was exempt from institutional review board approval.

### 2.1 The selection of IVs and the sources of data

Intake of beef, alcohol, non-oily fish, salad/raw vegetables, water, coffee, fresh fruit, oily fish, dried fruit, cereal, tea, salt, cooked vegetables, cheese, poultry, pork, lamb/mutton, bread, and processed meat were the dietary factors used as exposures in this study. The European-descent participants of dietary factors previously mentioned ranged from 335,394 to 462,630 individuals. The MRC Integrative Epidemiology Unit (IEU) at the University of Bristol funded the IEU open GWAS project, which either directly or indirectly extracted the GWAS data mentioned above from the UK Biobank. The GWAS data of Hernias (including umbilical, ventral, and inguinal hernias) were extracted from the FinnGen biobank. More information on the outcome and exposure datasets is provided in Supplementary Table S1 and Table 1. The IVs that were employed in the study were determined under the following criteria. First, we will immediately delete palindromic and missing SNPs. Second, linkage disequilibrium was at a level of r2 < 0.001, the threshold of genome-wide significance *p* < 5 × 10^−8^, and the clumping window at 10,000 kb. Third, the F statistics of the IVs must be higher than 10.

**Table 1:** Information on exposure and outcome datasets.

IEU GWAS id	Exposure or outcome	Participants included in analysis
ieu-b-73	Alcoholic drinks per week	335,394 European-descent individuals
ukb-b-5779	Alcohol intake frequency	462,346 European-descent individuals
ukb-b-6324	Processed meat intake	461,981 European-descent individuals
ukb-b-8006	Poultry intake	461,900 European-descent individuals
ukb-b-2862	Beef intake	461,053 European-descent individuals
ukb-b-17627	Non-oily fish intake	460,880 European-descent individuals
ukb-b-2209	Oily fish intake	460,443 European-descent individuals
ukb-b-5640	Pork intake	460,162 European-descent individuals
ukb-b-14179	Lamb/mutton intake	460,006 European-descent individuals
ukb-b-11348	Bread intake	452,236 European-descent individuals
ukb-b-1489	Cheese intake	451,486 European-descent individuals
ukb-b-8089	Cooked vegetable intake	448,651 European-descent individuals
ukb-b-6066	Tea intake	447,485 European-descent individuals
ukb-b-3881	Fresh fruit intake	446,462 European-descent individuals
ukb-b-15926	Cereal intake	441,640 European-descent individuals
ukb-b-1996	Salad / raw vegetable intake	435,435 European-descent individuals
ukb-b-5237	Coffee intake	428,860 European-descent individuals
ukb-b-16576	Dried fruit intake	421,764 European-descent individuals
ukb-b-8121	Salt added to food	462,630 European-descent individuals
ukb-b-14898	Water intake	427,588 European-descent individuals
finn-b-K11_UMBHER	Umbilical hernia	4,224 European-descent cases and 190,557 European-descent controls
finn-b-K11_VENTHER	Ventral hernia	3,737 European-descent cases and 190,557 European-descent controls
finn-b-K11_HERING	Inguinal hernia	17,096 European-descent cases and 190,557 European-descent controls

The information of the exposure and outcome datasets. More information about exposures and outcomes is available at the IEU OpenGWAS project (https://gwas.mrcieu.ac.uk/). IEU, Integrative Epidemiology Unit; GWAS, Genome-Wide Association Studies.

### 2.2 Statistical analysis

This study employed the inverse-variance weighted (IVW) method as the primary method for identifying causality. The IVW method, which requires that all SNPs remain valid or horizontal pleiotropy is balanced, offers the strongest power to identify causality (16). The weighted median method and the MR-Egger method were utilized as supplements to the IVW method, which served as the major method to assess causality in our study. If their results are consistent with the IVW method, the reliability of the IVW method will be greatly improved. Leave-one-out analysis was applied to evaluate the IVW method’s robustness. By employing the MR-Egger method, which allows for the existence of nonzero intercepts, the horizontal pleiotropy can be identified. To identify potential outliers, the MR-PRESSO method was used. Cochran’s Q test was utilized to assess heterogeneity. The R program (version 4.2.0) and TwoSampleMR package (17) were employed to perform all analyses.

## 3 Results

As shown in Supplementary Table S2, horizontal pleiotropy was detected in the analyses of the effects of fresh fruit intake on umbilical hernia and salt added to food on inguinal hernia (*p* < 0.05). The presence of horizontal pleiotropy indicated that these analyses violated the assumptions of MR and that there were direct associations between IVs and outcomes (18, 19). We will therefore treat them as invalid analyses. Utilizing the MR-PRESSO method, outliers were found in some analyses, but after removing the outliers and repeating the analyses, the results remained largely unchanged. The F statistics of IVs are all larger than 10, indicating that IVs and exposures have strong associations. The results of the MR-PRESSO method and F statistics are shown in the corresponding sections of Supplementary Table S2.

### 3.1 Dietary factors and inguinal hernia

**Figure 1:**
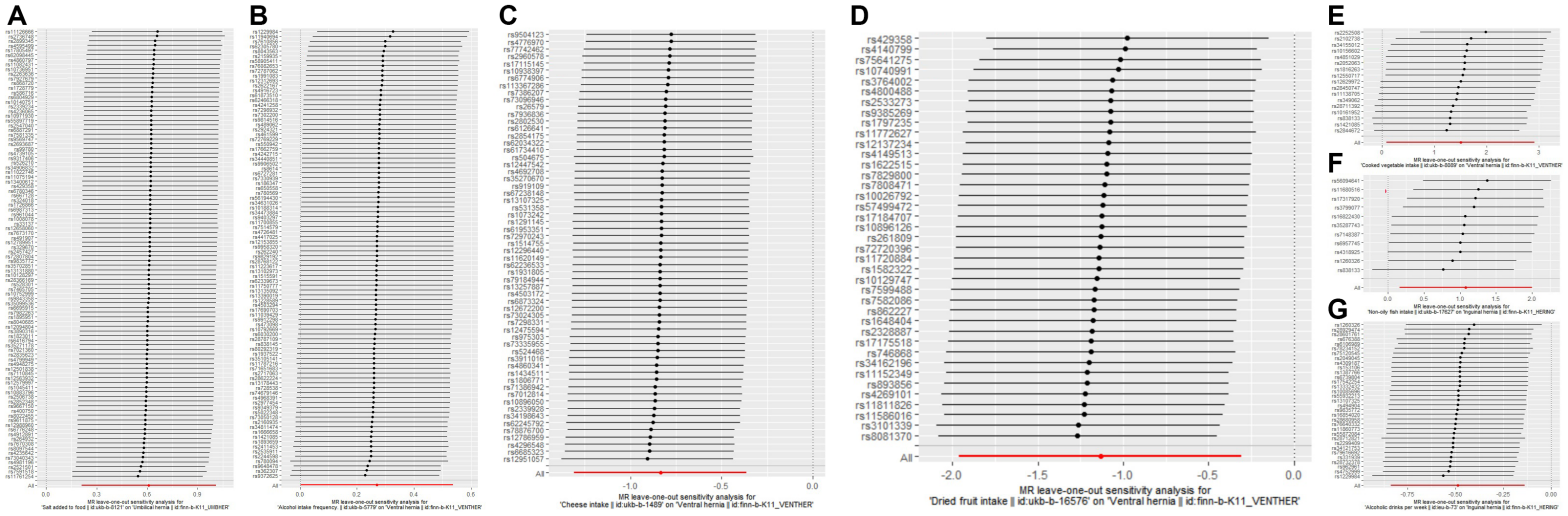
The results of leave-one-out analyses for **(A)** salt added to food on umbilical hernia **(B)** alcohol intake frequency on ventral hernia **(C)** cheese intake on ventral hernia **(D)** dried fruit intake on ventral hernia **(E)** cooked vegetable intake on ventral hernia **(F)** non-oily fish intake on inguinal hernia **(G)** alcoholic drinks per week on inguinal hernia.

Alcoholic drinks per week was observed to reduce the risk of inguinal hernia only in the IVW method (OR: 0.614; *p* = 0.00614). The intake of non-oily fish was observed to increase the risk of inguinal hernia in the IVW method (OR: 2.945; *p* = 0.0214) and the weighted median method (OR: 4.007; *p* = 0.0128). Lamb/mutton intake was observed to decrease the risk of inguinal hernia only in the MR-Egger method (OR: 0.0735; *p* = 0.0420). The MR-Egger method only complements the IVW method, so there is no causal relationship between lamb/mutton intake and inguinal hernia. Figure 1 shows the results of the leave-one-out analysis of positive dietary factors. Salt added to food is considered as an invalid analysis due to the detection of horizontal pleiotropy. Alcohol intake frequency and the intake of processed meat, poultry, beef, oily fish, pork, bread, cheese, cooked vegetable, tea, fresh fruit, cereal, salad/raw vegetable, coffee, dried fruit, and water were not associated with inguinal hernia in all of the three analysis methods (*p* > 0.05). More analysis results are provided in Supplementary Table S2.

### 3.2 Dietary factors and umbilical hernia

Salt added to food (cooking salt is not included) was observed to increase the risk of umbilical hernia only in the IVW method (OR: 1.841; *p* = 0.00267). Figure 1 shows the results of the leave-one-out analysis of positive dietary factors. Fresh fruit intake is considered as an invalid analysis due to the detection of horizontal pleiotropy. Horizontal pleiotropy was not detected in dried fruit intake after removal of an outlier. Cooked vegetable intake was observed to increase the risk of umbilical hernia only in the weighted median method (OR: 5.038; *p* = 0.0470). The weighted median method only complements the IVW method, so there is no causal relationship between cooked vegetable intake and umbilical hernia. The intake of alcohol, processed meat, non-oily fish, poultry, beef, oily fish, pork, lamb/mutton, bread, cheese, tea, cereal, salad/raw vegetable, coffee, dried fruit, and water were not associated with umbilical hernia in any of the three analysis methods (*p* > 0.05). More analysis results are provided in Supplementary Table S2.

### 3.3 Dietary factors and ventral hernia

It should be noted that the ventral hernia used in this study is defined according to the 10th revision of the International Classification of Diseases, mainly including incisional hernia and parastomal hernia (20).

Alcohol intake frequency was observed to increase the risk of ventral hernia only in the IVW method (OR: 1.309; *p* = 0.0477). Cheese intake and dried fruit intake were observed to decrease the risk of ventral hernia in the IVW method (cheese intake OR: 0.434; *p* = 0.000536; dried fruit intake OR: 0.322; *p* = 0.00716) and the weighted median method (cheese intake OR: 0.391; *p* = 0.00452; dried fruit intake OR: 0.239; *p* = 0.0107). Cooked vegetable intake was observed to increase the risk of ventral hernia in the IVW method (OR: 4.475; *p* = 0.0380) and the weighted median method (OR: 5.554; *p* = 0.0427). Figure 1 shows the results of the leave-one-out analysis of positive dietary factors. Non-oily fish intake was observed to increase the risk of ventral hernia only in the weighted median method (OR: 8.941; *p* = 0.0224). The weighted median method only complements the IVW method, so there is no causal relationship between cooked vegetable intake and umbilical hernia. Alcoholic drinks per week, salt added to food, and the intake of processed meat, poultry, beef, oily fish, pork, lamb/mutton, bread, tea, cereal, salad/raw vegetable, coffee, fresh fruit, and water were not associated with ventral hernia in the three analysis methods (*p* > 0.05). More analysis results are provided in Supplementary Table S2.

## 4 Discussion

The associations between nutritional factors and the risk of hernias have not been extensively studied (10), regardless of the fact that diet is an important influencing factor of health (21–23). We only found one study from Turkey that focused on the relationship between dietary factors and hernias, and their study found that dietary factors such as cheese, red meat, chicken, nuts, and bread were associated with inguinal hernia (10). Their study included only 115 people with groin and only used 3-day food consumption records; Therefore, we believe that their study has some limitations. Dietary factors are difficult to measure. The UK Biobank used the frequency to measure dietary factors, more information is provided in Supplementary Table S1. As changing eating habits is difficult, it is highly difficult to use randomized controlled trials to evaluate the effect of dietary factors on hernias. Observational epidemiology is often used to analyze the influence of research factors on study subjects. However, the presence of confounders (24–26), reverse causality (27, 28), and other factors might bias the causal effects that observational epidemiology observed. The introduction of instrumental variables can effectively solve these shortcomings (29, 30). MR, an analysis that uses genetic variation as IVs, is being utilized increasingly frequently. MR sits between observational epidemiology and interventional epidemiology in the hierarchy of evidence (31). In this study, we used MR analysis methods to analyze the effects of 20 dietary factors on three common hernias. The results of this study suggest that alcohol intake has different effects on different hernias. Alcoholic drinks per week reduce the risk of inguinal hernia, alcohol intake frequency increases the risk of abdominal hernia, and alcohol intake does not have any effect on umbilical hernia. In some studies, there was no association between alcohol intake and developing inguinal hernia (32, 33). However, it was found in a different study that individuals with inguinal hernias consumed more alcohol (10). However, less than 1,000 cases of inguinal hernias were included in their analysis, which limited the credibility of their study. Our study included hundreds of thousands of individuals from the UK Biobank and the Finngen Biobank, therefore our study provided new evidence to clarify the relationship between alcohol intake and inguinal hernia. It is worth noting that the causal relationship between drinking frequency and ventral hernia may be influenced by a single SNP, as shown in Figure 1B. In addition, we found that non-oily fish intake increased the risk of inguinal hernia, salt added to food increased the risk of umbilical hernia, cheese intake, and dried fruit intake reduced the risk of ventral hernia, and cooked vegetable intake increased the risk of ventral hernia. It is important to note that neither the causality of non-oily fish consumption on inguinal hernia nor the causality of cooked vegetable intake on ventral hernia are particularly stable; they are affected by a single SNP. More information is shown in Figures 1E,F. We must be particularly careful when interpreting these findings. First, the causal relationship observed by the MR analysis is the consequence of prolonged exposure to dietary factors. Therefore, short-term exposure may not have any clinical effect. Second, the Two-sample MR analysis only revealed the overall effects of exposures on outcomes, not the direct effects. Extremely complex pathways may link exposures and outcomes.

Unavoidably, this study has several restrictions. First, we were incapable of assessing whether there was a U-shaped correlation (for example, as dried fruit intake increases, the risk of ventral hernia rises first and then decreases) between dietary factors and hernias due to continuous data on dietary factors being employed in this study. Second, due to the lack of GWAS data for the two demographics of sex and age, we were unable to conduct stratified analyses. Third, the inability to further divide dietary intake categories prevents a more detailed analysis. Fourth, because our analysis primarily focuses on individuals from Europe, extending our findings to other populations is difficult.

## 5 Conclusion

Alcoholic drinks per week will reduce the risk of inguinal hernia, while alcohol intake frequency will not affect the risk of inguinal hernia. Alcohol intake frequency will increase the risk of ventral hernia, while alcoholic drinks per week will not affect the risk of ventral hernia. Alcohol intake will not affect the risk of umbilical hernia. The intake of non-oily fish will increase the risk of inguinal hernia. Salt added to food will increase the risk of umbilical hernia. Cheese intake and dried fruit intake will decrease the risk of ventral hernia, while cooked vegetable intake will increase the risk of ventral hernia. No causal relationships were found with hernias from other dietary factors.

## Data availability statement

The original contributions presented in the study are included in the article/Supplementary materials, further inquiries can be directed to the corresponding author/s.

## Ethics statement

Ethical approval was not required for the study involving humans in accordance with the local legislation and institutional requirements. Written informed consent to participate in this study was not required from the participants or the participants’ legal guardians/next of kin in accordance with the national legislation and the institutional requirements.

## Author contributions

YY: Data curation, Methodology, Formal analysis, Project administration, Validation, Funding acquisition, Resources, Visualization, Writing – original draft, Writing – review & editing. BH: Data curation, Methodology, Formal analysis, Project administration, Funding acquisition, Resources, Writing – original draft, Writing – review & editing. YW: Data curation, Methodology, Supervision, Conceptualization, Formal analysis, Project administration, Validation, Investigation, Funding acquisition, Resources, Visualization, Software, Writing – original draft, Writing – review & editing.

## References

[1] Dabbas N Adams K Pearson K Royle G. Frequency of abdominal wall hernias: is classical teaching out of date? JRSM Short Rep. (2011) 2:1–6. doi: 10.1258/shorts.2010.010071 21286228 PMC3031184

[2] Reistrup H Zetner DB Andresen K Rosenberg J. Prevention of incisional hernia[J]. Ugeskr Laeger. (2018) 180:V02180094. 30152315

[3] AhmedAlenazi A Alsharif MM Hussain MA. Prevalence, risk factors and character of abdominal hernia in Arar City, northern Saudi Arabia in 2017. Electron Physician. (2017) 9:4806–11. 28894539 10.19082/4806 PMC5586997

[4] Abramson JH Gofin J Hopp C Makler A Epstein LM. The epidemiology of inguinal hernia. A survey in western Jerusalem. J Epidemiol Community Health. (1978) 32:59–67. doi: 10.1136/jech.32.1.59, PMID: 95577 PMC1087312

[5] Akin ML Karakaya M Batkin A Nogay A. Prevalence of inguinal hernia in otherwise healthy males of 20 to 22 years of age. J R Army Med Corps. (1997) 143:101–2. doi: 10.1136/jramc-143-02-06, PMID: 9247863

[6] Sorensen LT Friis E Jorgensen T. Smoking is a risk factor for recurrence of groin hernia. World J Surg. (2002) 26:397–400. doi: 10.1007/s00268-001-0238-6, PMID: 11910469

[7] Gislason H Grønbech JE Søreide O. Burst abdomen and incisional hernia after major gastrointestinal operations–comparison of three closure techniques. Eur J. Surg. (1995) 161:349–54. PMID: 7662780

[8] Gislason H Søreide O Viste A. Wound complications after major gastrointestinal operations. The surgeon as a risk factor. Dig Surg. (1999) 16:512–4. doi: 10.1159/000018778 10805552

[9] Niggebrugge AH Trimbos JB Hermans J Steup WH Van De Velde CJ. Influence of abdominal-wound closure technique on complications after surgery: a randomised study. Lancet. (1999) 353:1563–7. doi: 10.1016/S0140-6736(98)10181-2 10334254

[10] Idiz C Cakir C. Nutritional status and constipation scoring of inguinal hernia patients: a case-control study. Hernia. (2020) 24:1107–12. doi: 10.1007/s10029-019-02075-8 31734784

[11] Cena H Calder PC. Defining a healthy diet: evidence for the role of contemporary dietary patterns in health and disease[J]. Nutrients. (2020) 12:334. doi: 10.3390/nu12020334, PMID: 32012681 PMC7071223

[12] Locke A Schneiderhan J Zick SM. Diets for health: goals and guidelines. Am Fam Physician. (2018) 97:721–8. PMID: 30215930

[13] Aggarwal M Bozkurt B Panjrath G Aggarwal B Ostfeld RJ Barnard ND. Lifestyle modifications for preventing and treating heart failure. J Am Coll Cardiol. (2018) 72:2391–405. doi: 10.1016/j.jacc.2018.08.2160 30384895

[14] Gumbs AA Gogol M Spolverato G Taher H Chouillard EK. Systematic review of the integrative medicine recommendations for patients with pancreatic Cancer. Surgeries. (2021) 2:216–30. doi: 10.3390/surgeries2020022

[15] Didelez V Sheehan N. Mendelian randomization as an instrumental variable approach to causal inference. Stat Methods Med Res. (2007) 16:309–30. doi: 10.1177/0962280206077743 17715159

[16] Hartwig FP Davey Smith G Bowden J. Robust inference in summary data Mendelian randomization via the zero modal pleiotropy assumption. Int J Epidemiol. (2017) 46:1985–98. doi: 10.1093/ije/dyx102, PMID: 29040600 PMC5837715

[17] Hemani G Zheng J Elsworth B. The MR-base platform supports systematic causal inference across the human phenome. eLife. (2018):7. 10.7554/eLife.34408 PMC5976434 29846171

[18] Davey Smith G Hemani G. Mendelian randomization: genetic anchors for causal inference in epidemiological studies. Hum Mol Genet. (2014) 23:R89–98. doi: 10.1093/hmg/ddu328, PMID: 25064373 PMC4170722

[19] Uo Bristol. Horizontal Pleiotropy. (2021-2022). Available at: https://mr-dictionary.mrcieu.ac.uk/term/horizontal-pleiotropy/

[20] Organization WH. the 10th revision of the International Classification of Diseases. (2016) Available at: https://icd.who.int/browse10/2016/en#/K43

[21] Tilman D Clark M. Global diets link environmental sustainability and human health. Nature. (2014) 515:518–22. doi: 10.1038/nature13959 25383533

[22] Touger-Decker R. Diet, cardiovascular disease and oral health: promoting health and reducing risk. J Am Dent Assoc. (1939) 141:167–70. doi: 10.14219/jada.archive.2010.0135 20123875

[23] Willett WC Sacks F Trichopoulou A Drescher G Ferro-Luzzi A Helsing E. Mediterranean diet pyramid: a cultural model for healthy eating. Am J Clin Nutr. (1995) 61:1402s–6s. doi: 10.1093/ajcn/61.6.1402S, PMID: 7754995

[24] Smith GD Ebrahim S. Data dredging, bias, or confounding. BMJ. (2002) 325:1437–8. doi: 10.1136/bmj.325.7378.1437, PMID: 12493654 PMC1124898

[25] Lawlor DA Davey Smith G Kundu D Bruckdorfer KR Ebrahim S. Those confounded vitamins: what can we learn from the differences between observational versus randomised trial evidence? Lancet. (2004) 363:1724–7. doi: 10.1016/S0140-6736(04)16260-0 15158637

[26] Taubes G. Epidemiology Faces Its Limits: The search for subtle links between diet, lifestyle, or environmental factors and disease is an unending source of fear—but often yields little certainty[J]. Science. (1995) 269, 164–169. 7618077 10.1126/science.7618077

[27] Fewell Z Davey Smith G Sterne JAC. The impact of residual and unmeasured confounding in epidemiologic studies: a simulation study. Am J Epidemiol. (2007) 166:646–55. doi: 10.1093/aje/kwm165, PMID: 17615092

[28] Sattar N Preiss D. Reverse causality in cardiovascular epidemiological research: more common than imagined? Circulation. (2017) 135:2369–72. doi: 10.1161/CIRCULATIONAHA.117.028307 28606949

[29] Widding-Havneraas T Zachrisson HD. A gentle introduction to instrumental variables. J Clin Epidemiol. (2022) 149:203–5. doi: 10.1016/j.jclinepi.2022.06.022, PMID: 35810980

[30] Greenland S. An introduction to instrumental variables for epidemiologists. Int J Epidemiol. (2000) 29:722–9. doi: 10.1093/ije/29.4.722, PMID: 10922351

[31] Zuccolo L Holmes MV. Commentary: Mendelian randomization-inspired causal inference in the absence of genetic data. Int J Epidemiol. (2017) 46:962–5. doi: 10.1093/ije/dyw327, PMID: 28025256

[32] Ruhl CE Everhart JE. Risk factors for inguinal hernia among adults in the US population. Am J Epidemiol. (2007) 165:1154–61. doi: 10.1093/aje/kwm011 17374852

[33] Carbonell JF Sanchez JL Peris RT. Risk factors associated with inguinal hernias: a case control study. Eur J Surg. (1993) 159:481–6. 8274556

