# Supplementary material for: Diet and risk for hernia: a Mendelian randomization analysis
Source: Front Nutr. 2024 Jun 18;11:1265920. doi: 10.3389/fnut.2024.1265920 (PMC11217535; doi:10.3389/fnut.2024.1265920)
Supplement: Supplementary file 2 [file Table_2.DOCX]

**Table 2 The results of Mendelian randomization analyses**

| outcome | IEU GWAS id | Exposure | F statistic | Used SNPs | inverse variance weighted method | | Weighted median method | | MR-Egger method | | Cochrane's Q test | | Pleiotropy | | | Outlier detection: the MR-PRESSO method | | | | | | | Outliers excluded | | | | | | | | | | |
| --- | --- | --- | --- | --- | --- | --- | --- | --- | --- | --- | --- | --- | --- | --- | --- | --- | --- | --- | --- | --- | --- | --- | --- | --- | --- | --- | --- | --- | --- | --- | --- | --- | --- |
|  |  |  |  |  | OR(95% CI) | P-value | OR(95% CI) | P-value | OR(95% CI) | P-value | Q | P-value | MR-Egger intercept | se | P-value | Raw | | | Outliers | outlier-corrected | | | inverse variance weighted method | | Weighted median method | | MR-Egger method | | Cochrane’s Q test | | Pleiotropy | | |
|  |  |  |  |  |  |  |  |  |  |  |  |  |  |  |  | casual estimate | sd | P-value |  | casual estimate | sd | P-value | OR(95% CI) | P-value | OR(95% CI) | P-value | OR(95% CI) | P-value | Q | P-value | MR-Egger intercept | se | P-value |
| Inguinal hernia | ieu-b-73 | Alcoholic drinks per week | 98.599 | 33 | 0.614(0.433-0.870) | 0.00614 | 0.690(0.421-1.130) | 0.140 | 0.578(0.259-1.293) | 0.192 | 40.314 | 0.149 | 0.00113 | 0.00701 | 0.873 | -0.488 | 0.178 | 0.00996 | N/A | N/A | N/A | N/A | N/A | N/A | N/A | N/A | N/A | N/A | N/A | N/A | N/A | N/A | N/A |
|  | ukb-b-5779 | Alcohol intake frequency | 115.259 | 92 | 1.081(0.923-1.267) | 0.332 | 1.152(0.933-1.422) | 0.189 | 1.350(0.829-2.198) | 0.230 | 141.364 | 0.000569 | -0.00558 | 0.00591 | 0.348 | 0.0783 | 0.0807 | 0.334 | rs56194430;rs58905411 | 0.0923 | 0.0726 | 0.206 | 1.097(0.951-1.264) | 0.203 | 1.162(0.948-1.423) | 0.147 | 1.420(0.922-2.188) | 0.115 | 109.141 | 0.0724 | -0.00649 | 0.00523 | 0.218 |
|  | ukb-b-6324 | Processed meat intake | 39.547 | 23 | 1.230(0.713-2.121) | 0.457 | 1.275(0.663-2.452) | 0.467 | 1.160(0.0704-19.114) | 0.918 | 39.503 | 0.0123 | 0.000880 | 0.0212 | 0.967 | 0.207 | 0.278 | 0.465 | N/A | N/A | N/A | N/A | N/A | N/A | N/A | N/A | N/A | N/A | N/A | N/A | N/A | N/A | N/A |
|  | ukb-b-8006 | Poultry intake | 24.411 | 7 | 1.116(0.435-2.866) | 0.819 | 1.177(0.327-4.245) | 0.803 | 9028.424(8.195e-10 - 9.946e+16) | 0.578 | 6.028 | 0.420 | -0.0974 | 0.166 | 0.582 | 0.110 | 0.481 | 0.827 | N/A | N/A | N/A | N/A | N/A | N/A | N/A | N/A | N/A | N/A | N/A | N/A | N/A | N/A | N/A |
|  | ukb-b-2862 | Beef intake | 27.841 | 14 | 1.019(0.514-2.020) | 0.957 | 1.330(0.544-3.250) | 0.532 | 0.793(0.0109-57.897) | 0.917 | 15.981 | 0.250 | 0.00319 | 0.0274 | 0.909 | 0.0187 | 0.349 | 0.958 | N/A | N/A | N/A | N/A | N/A | N/A | N/A | N/A | N/A | N/A | N/A | N/A | N/A | N/A | N/A |
|  | ukb-b-17627 | Non-oily fish intake | 27.544 | 11 | 2.945(1.174-7.393) | 0.0214 | 4.007(1.343-11.959) | 0.0128 | 13.227(0.143-1220.919) | 0.292 | 16.727 | 0.0806 | -0.0186 | 0.0280 | 0.523 | 1.080 | 0.470 | 0.0442 | N/A | N/A | N/A | N/A | N/A | N/A | N/A | N/A | N/A | N/A | N/A | N/A | N/A | N/A | N/A |
|  | ukb-b-2209 | Oily fish intake | 38.258 | 60 | 1.190(0.860-1.646) | 0.293 | 1.104(0.754-1.618) | 0.611 | 2.063(0.524-8.121) | 0.304 | 93.003 | 0.00313 | -0.00819 | 0.0101 | 0.421 | 0.174 | 0.165 | 0.298 | rs61882686 | 0.223 | 0.157 | 0.162 | 1.250(0.918-1.701) | 0.156 | 1.106(0.750-1.633) | 0.611 | 2.684(0.733-9.824) | 0.141 | 81.586 | 0.0223 | -0.0113 | 0.00955 | 0.240 |
|  | ukb-b-5640 | Pork intake | 18.071 | 13 | 0.703(0.246-2.005) | 0.510 | 0.449(0.145-1.390) | 0.165 | 7.476(0.00931-6007.223) | 0.567 | 22.914 | 0.0285 | -0.0245 | 0.0350 | 0.497 | -0.353 | 0.535 | 0.522 | rs838133 | -0.790 | 0.421 | 0.0872 | 0.454(0.199-1.035) | 0.0604 | 0.353(0.115-1.085) | 0.0691 | 2.252(0.0140-361.142) | 0.760 | 11.672 | 0.389 | -0.0165 | 0.0264 | 0.545 |
|  | ukb-b-14179 | Lamb/mutton intake | 19.720 | 30 | 0.741(0.408-1.346) | 0.324 | 0.791(0.349-1.793) | 0.575 | 0.0735(0.00665-0.811) | 0.0420 | 40.441 | 0.0770 | 0.0257 | 0.0132 | 0.0624 | -0.300 | 0.305 | 0.332 | N/A | N/A | N/A | N/A | N/A | N/A | N/A | N/A | N/A | N/A | N/A | N/A | N/A | N/A | N/A |
|  | ukb-b-11348 | Bread intake | 38.339 | 25 | 0.990(0.595-1.648) | 0.969 | 0.876(0.483-1.587) | 0.662 | 0.337(0.0316-3.595) | 0.377 | 41.012 | 0.0166 | 0.0156 | 0.0171 | 0.370 | -0.0101 | 0.260 | 0.969 | N/A | N/A | N/A | N/A | N/A | N/A | N/A | N/A | N/A | N/A | N/A | N/A | N/A | N/A | N/A |
|  | ukb-b-1489 | Cheese intake | 44.880 | 60 | 1.225(0.938-1.599) | 0.135 | 1.190(0.843-1.678) | 0.323 | 1.188(0.379-3.725) | 0.769 | 78.285 | 0.0473 | 0.000534 | 0.00980 | 0.957 | 0.203 | 0.136 | 0.141 | N/A | N/A | N/A | N/A | N/A | N/A | N/A | N/A | N/A | N/A | N/A | N/A | N/A | N/A | N/A |
|  | ukb-b-8089 | Cooked vegetable intake | 20.223 | 17 | 0.881(0.384-2.020) | 0.764 | 0.692(0.259-1.844) | 0.461 | 36.784(0.00365-3.712e+05) | 0.455 | 26.931 | 0.0423 | -0.0385 | 0.0484 | 0.438 | -0.127 | 0.424 | 0.768 | rs838133 | -0.436 | 0.364 | 0.250 | 0.647(0.317-1.321) | 0.232 | 0.528(0.199-1.399) | 0.199 | 0.331(6.795e-05 - 1616.128) | 0.802 | 17.118 | 0.312 | 0.00683 | 0.0441 | 0.879 |
|  | ukb-b-6066 | Tea intake | 62.782 | 39 | 1.150(0.902-1.466) | 0.258 | 1.229(0.837-1.806) | 0.292 | 1.063(0.625-1.808) | 0.822 | 36.738 | 0.528 | 0.00169 | 0.00517 | 0.746 | 0.140 | 0.122 | 0.257 | N/A | N/A | N/A | N/A | N/A | N/A | N/A | N/A | N/A | N/A | N/A | N/A | N/A | N/A | N/A |
|  | ukb-b-3881 | Fresh fruit intake | 15.502 | 52 | 0.658(0.422-1.028) | 0.0657 | 0.827(0.427-1.604) | 0.574 | 0.262(0.0580-1.180) | 0.0873 | 56.794 | 0.268 | 0.00887 | 0.00706 | 0.215 | -0.418 | 0.227 | 0.0716 | N/A | N/A | N/A | N/A | N/A | N/A | N/A | N/A | N/A | N/A | N/A | N/A | N/A | N/A | N/A |
|  | ukb-b-15926 | Cereal intake | 31.188 | 38 | 1.047(0.673-1.627) | 0.840 | 1.171(0.695-1.975) | 0.553 | 1.418(0.210-9.556) | 0.722 | 65.928 | 0.00239 | -0.00445 | 0.0139 | 0.750 | 0.0455 | 0.225 | 0.841 | rs10857964;rs838133 | 0.0910 | 0.207 | 0.663 | 1.095(0.730-1.644) | 0.661 | 1.427(0.853-2.386) | 0.176 | 3.608(0.624-20.871) | 0.161 | 47.510 | 0.0771 | -0.0171 | 0.0125 | 0.180 |
|  | ukb-b-1996 | Salad / raw vegetable intake | 17.571 | 18 | 1.473(0.598-3.629) | 0.400 | 0.865(0.311-2.406) | 0.781 | 0.514(0.00728-36.237) | 0.763 | 26.090 | 0.0728 | 0.0114 | 0.0230 | 0.626 | 0.388 | 0.460 | 0.411 | N/A | N/A | N/A | N/A | N/A | N/A | N/A | N/A | N/A | N/A | N/A | N/A | N/A | N/A | N/A |
|  | ukb-b-5237 | Coffee intake | 41.751 | 38 | 0.845(0.603-1.185) | 0.329 | 0.883(0.529-1.475) | 0.635 | 1.256(0.641-2.463) | 0.511 | 48.861 | 0.0918 | -0.00748 | 0.00563 | 0.192 | -0.168 | 0.172 | 0.335 | N/A | N/A | N/A | N/A | N/A | N/A | N/A | N/A | N/A | N/A | N/A | N/A | N/A | N/A | N/A |
|  | ukb-b-16576 | Dried fruit intake | 25.209 | 39 | 1.160(0.726-1.853) | 0.534 | 1.019( 0.560-1.853) | 0.952 | 2.429(0.301-19.610) | 0.410 | 63.681 | 0.00562 | -0.00923 | 0.0130 | 0.481 | 0.148 | 0.239 | 0.538 | N/A | N/A | N/A | N/A | N/A | N/A | N/A | N/A | N/A | N/A | N/A | N/A | N/A | N/A | N/A |
|  | ukb-b-8121 | Salt added to food | 36.232 | 96 | 0.877(0.705-1.092) | 0.242 | 0.888(0.650-1.213) | 0.454 | 1.853(0.900-3.818) | 0.0976 | 106.537 | 0.197 | -0.0111 | 0.00521 | 0.0363 | -0.131 | 0.112 | 0.245 | N/A | N/A | N/A | N/A | N/A | N/A | N/A | N/A | N/A | N/A | N/A | N/A | N/A | N/A | N/A |
|  | ukb-b-14898 | Water intake | 35.982 | 37 | 0.801(0.539-1.190) | 0.272 | 1.160(0.691-1.946) | 0.575 | 0.912(0.290-2.874) | 0.876 | 54.452 | 0.0249 | -0.00207 | 0.00872 | 0.814 | -0.222 | 0.202 | 0.279 | N/A | N/A | N/A | N/A | N/A | N/A | N/A | N/A | N/A | N/A | N/A | N/A | N/A | N/A | N/A |
| Umbilical hernia | ieu-b-73 | Alcoholic drinks per week | 98.599 | 33 | 1.539(0.843-2.809) | 0.160 | 0.919(0.394-2.145) | 0.845 | 1.394(0.345-5.634) | 0.644 | 36.560 | 0.265 | 0.00187 | 0.0121 | 0.878 | 0.431 | 0.307 | 0.170 | N/A | N/A | N/A | N/A | N/A | N/A | N/A | N/A | N/A | N/A | N/A | N/A | N/A | N/A | N/A |
|  | ukb-b-5779 | Alcohol intake frequency | 115.197 | 92 | 1.119(0.870-1.440) | 0.380 | 1.140(0.803-1.619) | 0.464 | 1.005(0.459-2.199) | 0.989 | 110.044 | 0.0850 | 0.00270 | 0.00949 | 0.777 | 0.1130 | 0.128 | 0.382 | N/A | N/A | N/A | N/A | N/A | N/A | N/A | N/A | N/A | N/A | N/A | N/A | N/A | N/A | N/A |
|  | ukb-b-6324 | Processed meat intake | 39.547 | 23 | 0.848(0.407-1.764) | 0.658 | 0.647(0.239-1.754) | 0.392 | 3.475(0.0880-137.149) | 0.514 | 13.255 | 0.926 | -0.0214 | 0.0279 | 0.451 | -0.165 | 0.290 | 0.575 | N/A | N/A | N/A | N/A | N/A | N/A | N/A | N/A | N/A | N/A | N/A | N/A | N/A | N/A | N/A |
|  | ukb-b-8006 | Poultry intake | 24.411 | 7 | 1.732(0.219-13.674) | 0.603 | 3.095(0.272-35.175) | 0.362 | 3.166e+05(2.181e-24 - 4.594e+34) | 0.727 | 8.893 | 0.180 | -0.131 | 0.371 | 0.738 | 0.549 | 1.054 | 0.621 | N/A | N/A | N/A | N/A | N/A | N/A | N/A | N/A | N/A | N/A | N/A | N/A | N/A | N/A | N/A |
|  | ukb-b-2862 | Beef intake | 27.841 | 14 | 0.490(0.149-1.617) | 0.241 | 0.756(0.151-3.786) | 0.733 | 2.036(0.00119-3476.691) | 0.855 | 14.930 | 0.312 | -0.0181 | 0.0476 | 0.710 | -0.713 | 0.609 | 0.263 | N/A | N/A | N/A | N/A | N/A | N/A | N/A | N/A | N/A | N/A | N/A | N/A | N/A | N/A | N/A |
|  | ukb-b-17627 | Non-oily fish intake | 27.544 | 11 | 3.726(0.821-16.908) | 0.0882 | 4.351(0.635-29.810) | 0.134 | 0.161(0.000108-241.724) | 0.636 | 13.886 | 0.178 | 0.0390 | 0.0453 | 0.412 | 1.315 | 0.772 | 0.119 | N/A | N/A | N/A | N/A | N/A | N/A | N/A | N/A | N/A | N/A | N/A | N/A | N/A | N/A | N/A |
|  | ukb-b-2209 | Oily fish intake | 38.088 | 60 | 1.637(0.965-2.779) | 0.0676 | 1.765(0.859-3.627) | 0.122 | 1.034(0.110-9.750) | 0.977 | 75.979 | 0.0676 | 0.0068 | 0.01660 | 0.681 | 0.493 | 0.270 | 0.0727 | N/A | N/A | N/A | N/A | N/A | N/A | N/A | N/A | N/A | N/A | N/A | N/A | N/A | N/A | N/A |
|  | ukb-b-5640 | Pork intake | 18.761 | 13 | 1.234(0.315-4.828) | 0.763 | 1.755(0.304-10.125) | 0.529 | 0.0370(7.511e-06 - 182.031) | 0.463 | 7.316 | 0.836 | 0.0364 | 0.0445 | 0.430 | 0.210 | 0.543 | 0.706 | N/A | N/A | N/A | N/A | N/A | N/A | N/A | N/A | N/A | N/A | N/A | N/A | N/A | N/A | N/A |
|  | ukb-b-14179 | Lamb/mutton intake | 19.720 | 30 | 0.739(0.297-1.839) | 0.516 | 1.114(0.305-4.067) | 0.870 | 0.281(0.00609-12.984) | 0.522 | 20.922 | 0.862 | 0.0107 | 0.0211 | 0.615 | -0.302 | 0.395 | 0.450 | N/A | N/A | N/A | N/A | N/A | N/A | N/A | N/A | N/A | N/A | N/A | N/A | N/A | N/A | N/A |
|  | ukb-b-11348 | Bread intake | 38.339 | 25 | 0.918(0.395-2.136) | 0.843 | 1.761(0.624-4.967) | 0.285 | 0.834(0.0153-45.525) | 0.930 | 34.599 | 0.075 | 0.00140 | 0.0289 | 0.962 | -0.0855 | 0.431 | 0.844 | N/A | N/A | N/A | N/A | N/A | N/A | N/A | N/A | N/A | N/A | N/A | N/A | N/A | N/A | N/A |
|  | ukb-b-1489 | Cheese intake | 44.880 | 60 | 0.675(0.426-1.068) | 0.0930 | 0.771(0.402-1.477) | 0.433 | 1.536(0.217-10.845) | 0.669 | 71.423 | 0.129 | -0.0142 | 0.0168 | 0.399 | -0.393 | 0.234 | 0.09830 | N/A | N/A | N/A | N/A | N/A | N/A | N/A | N/A | N/A | N/A | N/A | N/A | N/A | N/A | N/A |
|  | ukb-b-8089 | Cooked vegetable intake | 20.818 | 17 | 2.393(0.756-7.577) | 0.138 | 5.038(1.021-24.857) | 0.0470 | 0.000707(2.278e-09 - 219.772) | 0.279 | 15.642 | 0.478 | 0.0839 | 0.0664 | 0.225 | 0.872 | 0.582 | 0.153 | N/A | N/A | N/A | N/A | N/A | N/A | N/A | N/A | N/A | N/A | N/A | N/A | N/A | N/A | N/A |
|  | ukb-b-6066 | Tea intake | 62.782 | 39 | 1.157(0.717-1.869) | 0.550 | 1.466(0.742-2.898) | 0.271 | 1.164(0.402-3.366) | 0.781 | 45.599 | 0.185 | -0.000113 | 0.0103 | 0.991 | 0.146 | 0.245 | 0.553 | N/A | N/A | N/A | N/A | N/A | N/A | N/A | N/A | N/A | N/A | N/A | N/A | N/A | N/A | N/A |
|  | ukb-b-3881 | Fresh fruit intake | 15.502 | 52 | 0.795(0.361-1.751) | 0.569 | 0.490(0.144-1.662) | 0.252 | 0.0420(0.00316-0.559) | 0.0201 | 55.031 | 0.325 | 0.0283 | 0.0121 | 0.0239 | -0.230 | 0.403 | 0.571 | N/A | N/A | N/A | N/A | N/A | N/A | N/A | N/A | N/A | N/A | N/A | N/A | N/A | N/A | N/A |
|  | ukb-b-15926 | Cereal intake | 32.753 | 38 | 0.723(0.372-1.404) | 0.338 | 0.565(0.226-1.416) | 0.224 | 0.383(0.0218-6.726) | 0.516 | 45.944 | 0.149 | 0.00931 | 0.0208 | 0.658 | -0.324 | 0.339 | 0.345 | N/A | N/A | N/A | N/A | N/A | N/A | N/A | N/A | N/A | N/A | N/A | N/A | N/A | N/A | N/A |
|  | ukb-b-1996 | Salad / raw vegetable intake | 17.571 | 18 | 0.985(0.153-6.327) | 0.988 | 0.797(0.111-5.726) | 0.822 | 1.746(0.000258-11819.805) | 0.903 | 34.191 | 0.00793 | -0.00620 | 0.0476 | 0.898 | -0.0147 | 0.949 | 0.988 | N/A | N/A | N/A | N/A | N/A | N/A | N/A | N/A | N/A | N/A | N/A | N/A | N/A | N/A | N/A |
|  | ukb-b-5237 | Coffee intake | 41.751 | 38 | 1.387(0.798-2.409) | 0.246 | 1.448(0.671-3.124) | 0.345 | 1.552(0.503-4.785) | 0.449 | 40.179 | 0.331 | -0.00212 | 0.00941 | 0.823 | 0.327 | 0.282 | 0.253 | N/A | N/A | N/A | N/A | N/A | N/A | N/A | N/A | N/A | N/A | N/A | N/A | N/A | N/A | N/A |
|  | ukb-b-16576 | Dried fruit intake | 24.761 | 39 | 0.846(0.346-2.066) | 0.713 | 1.218(0.418-3.544) | 0.718 | 0.00794(0.000196-0.322) | 0.0147 | 71.358 | 0.000844 | 0.0583 | 0.0230 | 0.0156 | -0.168 | 0.456 | 0.715 | rs11772627 | 0.0871 | 0.441 | 0.845 | 1.091(0.459-2.591) | 0.844 | 1.570(0.548-4.499) | 0.401 | 0.0291(0.000595-1.427) | 0.0834 | 61.515 | 0.00689 | 0.0444 | 0.0238 | 0.0699 |
|  | ukb-b-8121 | Salt added to food | 36.232 | 96 | 1.841(1.236-2.742) | 0.00267 | 1.554(0.898-2.691) | 0.115 | 2.172(0.565-8.350) | 0.262 | 108.199 | 0.167 | -0.00245 | 0.00970 | 0.801 | 0.610 | 0.203 | 0.00341 | N/A | N/A | N/A | N/A | N/A | N/A | N/A | N/A | N/A | N/A | N/A | N/A | N/A | N/A | N/A |
|  | ukb-b-14898 | Water intake | 35.982 | 37 | 0.793(0.427-1.472) | 0.462 | 0.846(0.347-2.060) | 0.712 | 0.629(0.105-3.775) | 0.615 | 40.919 | 0.263 | 0.00368 | 0.0136 | 0.789 | -0.232 | 0.315 | 0.467 | N/A | N/A | N/A | N/A | N/A | N/A | N/A | N/A | N/A | N/A | N/A | N/A | N/A | N/A | N/A |
| Ventral hernia | ieu-b-73 | Alcoholic drinks per week | 98.599 | 33 | 1.016(0.472-2.188) | 0.967 | 1.842(0.737-4.604) | 0.192 | 2.060(0.358-11.858) | 0.425 | 53.413 | 0.0102 | -0.0134 | 0.0152 | 0.385 | 0.0162 | 0.391 | 0.967 | N/A | N/A | N/A | N/A | N/A | N/A | N/A | N/A | N/A | N/A | N/A | N/A | N/A | N/A | N/A |
|  | ukb-b-5779 | Alcohol intake frequency | 115.197 | 92 | 1.309(1.003-1.710) | 0.0477 | 1.393(0.943-2.058) | 0.0960 | 0.676(0.299-1.527) | 0.348 | 110.752 | 0.0780 | 0.0166 | 0.00988 | 0.0962 | 0.270 | 0.136 | 0.0507 | N/A | N/A | N/A | N/A | N/A | N/A | N/A | N/A | N/A | N/A | N/A | N/A | N/A | N/A | N/A |
|  | ukb-b-6324 | Processed meat intake | 39.547 | 23 | 1.710(0.767-3.812) | 0.190 | 1.312(0.433-3.969) | 0.631 | 55.359(1.145-2677.655) | 0.0554 | 23.566 | 0.370 | -0.0528 | 0.0294 | 0.0873 | 0.536 | 0.409 | 0.204 | N/A | N/A | N/A | N/A | N/A | N/A | N/A | N/A | N/A | N/A | N/A | N/A | N/A | N/A | N/A |
|  | ukb-b-8006 | Poultry intake | 24.411 | 7 | 3.559(0.592-21.380) | 0.165 | 3.530(0.317-39.332) | 0.305 | 1.89e+11(7.969e-13 - 4.493e+34) | 0.388 | 4.088 | 0.665 | -0.267 | 0.297 | 0.409 | 1.269 | 0.755 | 0.144 | N/A | N/A | N/A | N/A | N/A | N/A | N/A | N/A | N/A | N/A | N/A | N/A | N/A | N/A | N/A |
|  | ukb-b-2862 | Beef intake | 27.841 | 14 | 1.668(0.352-7.904) | 0.519 | 1.378(0.246-7.716) | 0.716 | 0.0165(1.375e-06 - 197.928) | 0.408 | 22.665 | 0.0459 | 0.0586 | 0.0600 | 0.348 | 0.512 | 0.794 | 0.530 | N/A | N/A | N/A | N/A | N/A | N/A | N/A | N/A | N/A | N/A | N/A | N/A | N/A | N/A | N/A |
|  | ukb-b-17627 | Non-oily fish intake | 27.544 | 11 | 3.418(0.738-15.843) | 0.116 | 8.941(1.364-58.626) | 0.0224 | 4057.586(6.295-2.615e+06) | 0.0329 | 12.826 | 0.234 | -0.0879 | 0.0401 | 0.0559 | 1.229 | 0.782 | 0.147 | N/A | N/A | N/A | N/A | N/A | N/A | N/A | N/A | N/A | N/A | N/A | N/A | N/A | N/A | N/A |
|  | ukb-b-2209 | Oily fish intake | 38.088 | 60 | 1.452(0.865-2.437) | 0.159 | 1.597(0.766-3.328) | 0.212 | 3.508(0.394-31.242) | 0.265 | 65.426 | 0.264 | -0.0131 | 0.0161 | 0.419 | 0.373 | 0.264 | 0.164 | N/A | N/A | N/A | N/A | N/A | N/A | N/A | N/A | N/A | N/A | N/A | N/A | N/A | N/A | N/A |
|  | ukb-b-5640 | Pork intake | 18.761 | 13 | 2.313(0.511-10.469) | 0.276 | 3.742(0.489-28.627) | 0.204 | 0.346(1.989e-05 - 6001.353) | 0.835 | 13.165 | 0.357 | 0.0197 | 0.0510 | 0.706 | 0.839 | 0.770 | 0.298 | N/A | N/A | N/A | N/A | N/A | N/A | N/A | N/A | N/A | N/A | N/A | N/A | N/A | N/A | N/A |
|  | ukb-b-14179 | Lamb/mutton intake | 19.720 | 30 | 0.677(0.222-2.069) | 0.494 | 0.788(0.186-3.344) | 0.746 | 27.142(0.279-2643.088) | 0.169 | 38.975 | 0.102 | -0.0410 | 0.0252 | 0.115 | -0.390 | 0.570 | 0.499 | N/A | N/A | N/A | N/A | N/A | N/A | N/A | N/A | N/A | N/A | N/A | N/A | N/A | N/A | N/A |
|  | ukb-b-11348 | Bread intake | 38.339 | 25 | 1.085(0.488-2.414) | 0.841 | 0.912(0.330-2.524) | 0.859 | 8.147(0.203-327.192) | 0.277 | 27.766 | 0.270 | -0.0293 | 0.0267 | 0.285 | 0.0817 | 0.408 | 0.843 | N/A | N/A | N/A | N/A | N/A | N/A | N/A | N/A | N/A | N/A | N/A | N/A | N/A | N/A | N/A |
|  | ukb-b-1489 | Cheese intake | 44.880 | 60 | 0.434(0.271-0.696) | 0.000536 | 0.391(0.204-0.747) | 0.00452 | 0.459(0.0605-3.475) | 0.454 | 67.765 | 0.203 | -0.000960 | 0.0174 | 0.956 | -0.835 | 0.241 | 0.00100 | N/A | N/A | N/A | N/A | N/A | N/A | N/A | N/A | N/A | N/A | N/A | N/A | N/A | N/A | N/A |
|  | ukb-b-8089 | Cooked vegetable intake | 20.818 | 17 | 4.475(1.087-18.427) | 0.0380 | 5.554(1.058-29.152) | 0.0427 | 66.840(7.560e-06 - 5.910e+08) | 0.614 | 21.599 | 0.157 | -0.0279 | 0.0839 | 0.744 | 1.498 | 0.722 | 0.0545 | N/A | N/A | N/A | N/A | N/A | N/A | N/A | N/A | N/A | N/A | N/A | N/A | N/A | N/A | N/A |
|  | ukb-b-6066 | Tea intake | 62.782 | 39 | 1.305(0.822-2.071) | 0.258 | 1.913(0.945-3.871) | 0.0714 | 1.547(0.560-4.270) | 0.405 | 37.561 | 0.490 | -0.00364 | 0.00989 | 0.715 | 0.266 | 0.234 | 0.263 | N/A | N/A | N/A | N/A | N/A | N/A | N/A | N/A | N/A | N/A | N/A | N/A | N/A | N/A | N/A |
|  | ukb-b-3881 | Fresh fruit intake | 15.502 | 52 | 0.679(0.304-1.517) | 0.345 | 0.959(0.291-3.160) | 0.945 | 1.733(0.113-26.588) | 0.695 | 49.748 | 0.523 | -0.00902 | 0.0128 | 0.485 | -0.387 | 0.405 | 0.344 | N/A | N/A | N/A | N/A | N/A | N/A | N/A | N/A | N/A | N/A | N/A | N/A | N/A | N/A | N/A |
|  | ukb-b-15926 | Cereal intake | 32.753 | 38 | 0.524(0.254-1.081) | 0.0802 | 0.466(0.185-1.178) | 0.107 | 0.113(0.00512-2.504) | 0.176 | 49.035 | 0.0891 | 0.0224 | 0.0225 | 0.325 | -0.647 | 0.370 | 0.0885 | N/A | N/A | N/A | N/A | N/A | N/A | N/A | N/A | N/A | N/A | N/A | N/A | N/A | N/A | N/A |
|  | ukb-b-1996 | Salad / raw vegetable intake | 17.585 | 18 | 0.892(0.113-7.060) | 0.914 | 0.294(0.0370-2.331) | 0.246 | 18.076(0.00107-3.050e+05) | 0.568 | 37.994 | 0.00246 | -0.0326 | 0.0525 | 0.544 | -0.114 | 1.055 | 0.915 | rs3129962 | -0.472 | 0.939 | 0.622 | 0.624(0.0991-3.928) | 0.615 | 0.284(0.0370-2.173) | 0.225 | 3.404(0.000543-21335.668) | 0.787 | 27.607 | 0.0352 | -0.0183 | 0.0470 | 0.702 |
|  | ukb-b-5237 | Coffee intake | 41.751 | 38 | 1.730(0.896-3.341) | 0.103 | 2.135(0.956-4.770) | 0.0644 | 3.448(0.924-12.872) | 0.0737 | 51.034 | 0.0622 | -0.0130 | 0.0110 | 0.245 | 0.548 | 0.336 | 0.111 | N/A | N/A | N/A | N/A | N/A | N/A | N/A | N/A | N/A | N/A | N/A | N/A | N/A | N/A | N/A |
|  | ukb-b-16576 | Dried fruit intake | 25.209 | 39 | 0.322(0.141-0.735) | 0.00716 | 0.239(0.0794-0.717) | 0.0107 | 0.425(0.0104-17.422) | 0.654 | 54.624 | 0.0394 | -0.00346 | 0.0231 | 0.881 | -1.134 | 0.422 | 0.0106 | N/A | N/A | N/A | N/A | N/A | N/A | N/A | N/A | N/A | N/A | N/A | N/A | N/A | N/A | N/A |
|  | ukb-b-8121 | Salt added to food | 34.989 | 96 | 1.551(0.978-2.459) | 0.0621 | 1.178(0.641-2.165) | 0.598 | 1.948(0.412-9.221) | 0.403 | 129.841 | 0.0102 | -0.00337 | 0.0112 | 0.764 | 0.439 | 0.235 | 0.0652 | rs9611875 | 0.323 | 0.230 | 0.163 | 1.381(0.881-2.167) | 0.159 | 1.084(0.576-2.039) | 0.803 | 0.577(0.108-3.074) | 0.521 | 118.922 | 0.0422 | 0.0126 | 0.0119 | 0.291 |
|  | ukb-b-14898 | Water intake | 35.982 | 37 | 0.571(0.281-1.160) | 0.121 | 0.453(0.181-1.138) | 0.0921 | 0.302(0.0392-2.330) | 0.259 | 48.071 | 0.0860 | 0.0101 | 0.0155 | 0.519 | -0.560 | 0.362 | 0.130 | N/A | N/A | N/A | N/A | N/A | N/A | N/A | N/A | N/A | N/A | N/A | N/A | N/A | N/A | N/A |

The heterogeneity was measured employing the Cochran's Q test. The MR-PRESSO method was employed to identify possible outliers. Once any outliers are detected, we repeat all Mendelian randomization analyses and present the results in the ‘Outliers excluded’ section. If the ‘Outliers excluded’ section shows "N/A", no outliers were found. the inverse-variance-weighted (IVW) method is the primary method for identifying causality.

OR: Odds ratio; SNPs: Single-nucleotide polymorphisms; CI: Confidence interval; NA: Not available;
